# Psychometric properties and item response theory analysis of the Persian version of the social pain questionnaire

**DOI:** 10.3389/fpsyg.2024.1372229

**Published:** 2024-04-12

**Authors:** Mahya Sepehrinia, Hojjatollah Farahani, Peter Watson, Nasim Amini

**Affiliations:** ^1^Department of Psychology, Tarbiat Modares University, Tehran, Iran; ^2^MRC Cognition and Brain Sciences Unit, University of Cambridge, Cambridge, United Kingdom; ^3^Department of Psychology, University of Mohaghegh Ardabili, Ardabil, Iran

**Keywords:** social pain, ostracism, social exclusion, rejection, psychometric, IRT, reliability, validity

## Abstract

**Introduction:**

Social pain is an emotional reaction which is triggered by social exclusion and has been extensively investigated in the literature. The Social Pain Questionnaire (SPQ) is a self-report instrument which is the only scale for measuring social pain as a dispositional factor. The current study aimed at examining the psychometric properties of the SPQ in an Iranian sample.

**Materials and methods:**

A sample of participants (*N* = 400) was recruited in a cross-sectional validation study. Exploratory Factor Analysis (EFA) as well as Confirmatory Factor Analysis (CFA) were conducted. The Item Response Theory (IRT) model parameters were evaluated and item response category curves were presented. Convergent and divergent validities as well as the reliability (by using Cronbach’s alpha coefficient) were also assessed.

**Results:**

The SPQ’s unidimensionality was affirmed (RMSEA = 0.078; CFI = 0.915; TLI = 0.99) and its internal consistency was robust (Cronbach’s α = 0.94). The correlation between the SPQ and the following measures endorsed its divergent and convergent validity: Self-esteem (*r* = −0.424), Perceived Social Support (*r* = −0.161), and Interpersonal Sensitivity (*r* = 0.636). Finally, Item Response Theory Analysis emphasized the effectiveness of the SPQ items in discerning various levels of social pain. The theta level ranged between −1 and + 1.2 and the IRT-based marginal reliability was 0.92 for the total score.

**Discussion:**

The Persian SPQ stands as a reliable and valid measure for evaluating social pain. This scale has the potential to stimulate further research in the field for both clinical and non-clinical settings.

**Conclusion:**

By employing Item Response Theory (IRT) analysis, we have transcended the theoretical psychometric evaluation of the SPQ scale and demonstrated that SPQ is a unidimensional, valid and reliable measurement tool.

## Introduction

Social pain refers to a specific emotional reaction and is defined as “the perception that one is being excluded from desired relationships or being devalued by desired relationship partners or groups.” (MacDonald and Leary, 2005, p. 202). Exclusion occurs in daily experience resulting from various factors, including rejection [e.g., when opinions are ignored in a work meeting or over the telephone, mail, email, or chatroom (Nezlek et al., 2012)] or loss of a loved one, either by death or separation (MacDonald and Leary, 2005). Moreover, prolonged exclusion results in other negative feelings like helplessness, alienation, depression, and unworthiness (Hartgerink et al., 2015) therefore, social exclusion can be associated with lower levels of psychological well-being (De Ruddere et al., 2016). The common and distressful experience of social pain resulting from exclusion has been an issue of concern in the literature (Poon et al., 2020).

According to (the evolutionary basis of) the social pain theory (MacDonald and Leary, 2005), separation from important social entities and loss of social connections were dangerous and presented significant challenges to our ancestors’ survival (Baumeister and Leary, 1995; Wirth et al., 2020). The most likely social animals to survive and raise offspring to reproductive age were those that had strong close relationships and were the most integrated into group living (Baumeister and Leary, 1995). Human survival, thus, depends on social acceptance and social inclusion (DeWall, 2013) and in the long term, social exclusion can lead to not only being excluded from society but also to premature death (Williams and Nida, 2011). In this context, social pain alerts people about exclusion and entices the individual to restore social relationships (Wirth et al., 2020) moreover because of the vital role of social pain in human survival people take social pain seriously (MacDonald and Leary, 2005).

Social and physical pain share similar underlying mechanisms in terms of survival functions and physiological responses (MacDonald and Leary, 2005; Woo et al., 2014; Eisenberger, 2015). Based on the social pain theory, the survival function of social pain is similar to that of physical pain. More precisely, both pains lead animals away from threatening situations and towards helpful people. In other words, physical and social pain serve as a warning signal that a harmful or damaging event has occurred and trigger generalized threat-response mechanisms (MacDonald and Leary, 2005). In addition according to the social-physical overlap theory (Eisenberger et al., 2003) social and physical pain share common neural and neurochemical substrates such as the dorsal anterior cingulate cortex (dACC) (Eisenberger, 2015; Rotge et al., 2015; Zhang et al., 2019). Other studies criticized this theory and showed that even though the physical pain and social pain may activate similar brain areas they have distinct and uncorrelated fMRI patterns within pain processing regions (Woo et al., 2014) and the default network not dACC is the key region involved in the experience of social pain (Mwilambwe-Tshilobo and Spreng, 2021). Consistent with the distinct representation of physical and social pain, these two pains are re-experienced differently. The reliving and re-experience of social pain is easier and more intense than physical pain. In addition, after experiencing social pain, the performance on cognitively demanding tasks decreases more after experiencing physical pain (Chen et al., 2008).

While a variety of measures have been employed within the extant literature of social pain (Hudd and Moscovitch, 2020), the needs-threat scale has been extensively used as the basis to explain emotional responses of social pain to social exclusion. Specifically, according to the need-threat model (Williams, 2001), as a result of social exclusion, four basic needs are threatened including belongingness, maintaining high self-esteem, perceiving personal control, and existing meaning. Perceiving threat, therefore, to each of these four motives generates aggressive or prosocial behaviors. To investigate social pain based on the needs-threat model, the Cyberball paradigm is commonly used to experimentally manipulate social exclusion (Hartgerink et al., 2015). Even though the paradigm has internal validity and reliability its external validity is questioned (Wirth, 2016). Specifically, the emotional reactions to being socially excluded in the Cyberball game are limited to a specific condition and cannot be generalized to other conditions (Wirth, 2016; Stangier et al., 2021). In addition, the needs-threat scale only assesses short-term emotional reactions as a response to being socially excluded not as a long-term feeling of pain (Stangier et al., 2021). Other studies employ different measures; for example, the Negative Affect (NA) subscale of the Positive and Negative Affect Schedule (Auyeung and Alden, 2016), the Hurt Feelings Scale (Slavich et al., 2019), and the McGill Pain Questionnaire (Chester et al., 2016) but none of these has been well-validated as a distinct measure of social pain.

Relying on physical pain overlap theory (Eisenberger et al., 2003) and belongingness theory (Baumeister and Leary, 1995), Stangier et al. (2021) proposed the first scale to assess social pain as a response predisposition due to the lack of standardized and validated instruments related to it. The final version of Social Pain questionnaire (SPQ) consists of 10 items, constituting a solid one-factor structure construct, achieved after a three-step procedure including development of an item pool (where 46 statements were selected), first item reduction (leaving 18 items), and second item reduction (leaving 10 items). In terms of factor structure, reliability, convergent validity, and discriminant validity, the Social Pain Questionnaire has robust psychometric properties. In order to check the factorial structure of the questionnaire, confirmatory factor analysis (CFA) was conducted on the online sample (*n* = 623), the representative sample (*n* = 2,531) and the patient sample (*n* = 270) (Stangier et al., 2021). Despite providing researchers with a valuable measure there are several limitations that need to be addressed in order to confirm and extend the original findings as well as refine the measure to improve its psychometric properties. First, Stangier et al. (2021) examined the factor structure of the SPQ only by conducting a CFA, though it is also necessary to conduct Exploratory Factor Analysis (EFA) to confirm the CFA-derived unidimensional factor structure of the SPQ. Second, in order to evaluate the latent trait of social pain across its entire severity range, Item Response Theory (IRT) models could provide further information about the scale. In IRT models, the latent trait is estimated using observed variables and related to item characteristics (difficulty, discrimination, and individual levels) and likelihoods of selecting various responses within the item (De Ayala and Little, 2022). However, IRT analysis has not been applied to the SPQ. Third, SPQ scores must also be examined in relation to other external correlates of interests such as interpersonal sensitivity, self-esteem, and perceived social support in order to further confirm their validity. Fourth, this scale has not been studied among different cultures and societies. By investigating the psychometric properties of the SPQ in an Eastern country (i.e., Iran) it is possible to assess whether any modifications are needed to enhance the validity, reliability, and factor structure of the measure in different cultural contexts. Finally, Iran does not yet have a measure suitable for assessing the experience of social pain. In order to investigate social pain for Iranian researchers it follows that established measures of social pain need to be translated and evaluated.

### Present study

This study was conducted in an effort to validate the Persian version of the SPQ. We aim to explore the psychometric properties of the Persian Social Pain Questionnaire by measuring its internal consistency, reliability, and construct validity (including convergent and divergent validity) in a nonclinical sample of Iranians. To establish convergent validity, we examine the association of the SPQ with the Interpersonal Sensitivity Measure (Boyce and Parker, 1989). Both social pain and interpersonal sensitivity involve a loss of a protective social bond (Eisenberger, 2012) with a moderate to high positive correlation expected between them. The self-esteem scale of Rosenberg (1965) was used to determine divergent validity. Based on the sociometer theory Leary and Terry (2013) state that self-esteem refers to the degree to which individuals perceive themselves to be valued and accepted by others in the near future. Social exclusion undermines the importance of self-esteem as a psychological need (Williams, 2009; Williams and Nida, 2011), thus in line with previous studies (Yanagisawa et al., 2011), it is plausible to assume that self-esteem has a negative relationship with social pain. We also used the Perceived Social Support scale (Zimet et al., 1988) in order to further evaluate the divergent validity of SPQ. We hypothesize that there will be a negative association between Social Pain and Perceived Social Support, due to the importance of needing to have frequent and positive relationships with supportive others (Masten et al., 2012). Finally, in this study Item Response Theory (IRT) analysis was performed to evaluate the difficulty and discrimination parameters of each of the SPQ items.

## Method

### Participants

It is recommended that for loadings of at least 0.3 with one factor a sample size of 120 participants is required when conducting Confirmatory Factor Analysis (CFA) in order to reliably estimate the model parameters (Jackson, 2003). In this study, a large sample of 400 individuals was recruited to enhance statistical power. Participants ranged in age from 18 to 73 years old (*M* = 32.29, SD = 9.59), 74.5% were female with no difference in mean age between men and women The majority of participants (63.5%) were single. 9.8% of the aggregated sample were pursuing or already held a high school diploma, 35.5% held a bachelor’s degree, 43.3% had gained a master’s degree, and 11.5% had a doctorate. From August to October 2023 data were collected with participants recruited through convenience sampling.

Invitations to participate in the study were issued to Iranian adults via multiple online social media platforms, such as Twitter, WhatsApp, Instagram, and Telegram. The survey was placed on the online platform of Google Forms. According to the inclusion criteria, participants were required to be adults (age > 18) who were willing to participate. Participants returning incomplete questionnaires were excluded. Those interested in participating affirmed their involvement using an online informed consent form where there was a brief explanation of the main purpose of the study. All the participants received guarantees regarding the security and confidentiality of their data.

### Translation process

The original English version of the SPQ was firstly translated into Persian. Two English-Persian translators, who were expert in the study’s domain, then reviewed and discussed this translation of the questionnaire. These researchers then modified the initial translation. The Persian translation which was made in the previous stage was now translated back into English by a bi-lingual speaker of Persian and English. An expert compared the results of this back-translation into English with the original English version of the SPQ. The two versions were found to be in agreement and no further modifications were, therefore, required.

### Measures

#### Social pain questionnaire

The SPQ (Stangier et al., 2021) is a self-report measure consisting of 10 items. This scale involves items regarding the perception of social pain (e.g., It hurts me when somebody ignores me). Participants rate the items on a 5-point scale from 0 (*applies exactly to me*) to 4 (*applies not at all to me*). In terms of internal consistency, the questionnaire has been found to be highly reliable (Cronbach’s α = 0.94) (Stangier et al., 2021). The convergent validity of the scale has been demonstrated by finding sizeable correlations with the Interpersonal Sensitivity Measure (IPSM) (*r* = 0.69), anxious ambivalent attachment styles (*r* = 0.5), depression (*r* = 0.3), and social anxiety (*r* = 0.68). Additionally, the divergent validity of the scale was confirmed by the lack of a correlation with numbers of somatic complaints (*r* = 0.18) (Schwarz et al., 2021).

#### Interpersonal sensitivity measure

The original version of the measure was developed by Boyce and Parker (1989). The IPSM assesses how individuals are sensitive to the behaviors of others, feedback and negative evaluations of a personal nature. IPSM consists of 36 items on a four-point Likert scale, ranging from 1 (*very like you*) to 4 (*very unlike you*). The internal consistency of the scale’s total score was high in both nonclinical (Cronbach’s α = 0.86) and depressed patients (Cronbach’s α = 0.85). The IPSM involves five subscales including; interpersonal awareness, need for approval, separation anxiety, timidity, and fragile inner-self. Cronbach’s alpha coefficients for these subscales were 0.76, 0.55, 0.67, 0.63, and 0.59, respectively. Reliability of the scale was 0.70 and assessed using test–retest correlations over a 6-week period. The Persian version of IPSM was created by Mohammadian et al. (2017) and showed test–retest correlations of 0.86 for total score and Cronbach’s alphas of 0.70, 0.51, 0.58, 0.58, and 0.70 for the respective subscales. Test–retest reliability among the Iranian sample was high with correlations ranging from 0.73 to 0.92, over a 2-week period.

#### Self-esteem scale

The self-esteem scale is a single common construct originating from Rosenberg (1965) with the Persian version developed by Shapurian et al. (1987). The scale consists of 10 items each scored on a four-point Likert scale, ranging from 1 (*Strongly Agree*) to 4 (*Strongly Disagree*). The original version of the Self-esteem scale showed high internal consistency (Rosenberg, 1979; Neria, 2016) and the Persian version of the scale also indicated good test–retest reliability over 3 weeks (Cronbach’s α = 0.84), and internal consistency (Cronbach’s α for sample I was equal to 0.82 and for sample II it was equal to 0.83) (Shapurian et al., 1987).

#### Multidimensional scale of perceived social support (MSPSS)

The original English version of the MSPSS was developed by Zimet et al. (1988) and measures participants’ perception of support from family members, friends, and significant others. The Persian version of the MSPSS was developed by Bagherian-Sararoudi et al. (2013). The MSPSS consists of 12 items and involves subscales including family, friends, and significant others. Items are rated on a seven-point Likert scale ranging from 0 (*very strongly disagree*) to 6 (*very strongly agree*). In a healthy group, test–retest reliability over 2-weeks was good with correlations of 0.74, 0.78, and 0.84 for family, friends and significant others, respectively. Internal consistency of the items was also high with a Cronbach’s α of 0.92 (Bagherian-Sararoudi et al., 2013).

### Statistical analyses

The psychometric properties of the SPQ scale were examined in several stages. First, ceiling and floor effects were checked for using a cut-off point of 15%, as recommended by Terwee et al. (2007). We evaluated the construct validity of the Persian version of the SPW using confirmatory factor analysis (CFA) and exploratory factor analysis (EFA). The Kaiser–Meyer–Olkin test (KMO > 0.7 is acceptable) and Bartlett’s test of sphericity (A value of *p* < 0.05 is acceptable) were conducted to evaluate the adequacy of sampling and suitability of the sample (Kaiser, 1974). Items with loadings having an absolute value of 0.3 or greater were considered as loading on a factor. The Lavaan (Chalmers, 2012) package in RStudio version 4.2. was used to test the fit of the originally proposed unidimensional factor structure of the SPQ. Model fit was assessed using several indices, including χ^2^ and its degrees of freedom (*p* > 0.05), the comparative fit index (CFI ≥ 0.90; Bentler and Bonett, 1980), Tucker–Lewis Index (TLI ≥ 0.90; Hu and Bentler, 1999), and the root mean square error of approximation (RMSEA) with values less than 0.08 indicating a fair fit (Browne and Cudeck, 1992),

Cronbach’s alpha was used in order to calculate internal consistency with a threshold over 0.7 considered acceptable (DeVellis and Thorpe, 2022). Finally, correlations between the IPSM and the SPQ were used to measure the construct validity of the SPQ while correlations between Self-esteem, MSPSS, and SPQ were evaluated to measure its divergent validity. All analyses were conducted in IBM® SPSS® 26 (Statistical Package for the Social Sciences) unless otherwise specified. A *p*-value of <0.05 was considered as the threshold for statistical significance.

The IRT analysis was conducted using the mirt (Rosseel, 2012) package in RStudio version 4.2. Prior to conducting the analysis, we checked the assumption of unidimensionality. In addition to CFA, we also performed an exploratory factor analysis to assess whether there was a unidimensional structure. To evaluate dimensionality, we followed the recommendation that the first-to-second eigenvalue ratio should be greater than 3 for unidimensionality to be established. After assessing dimensionality, we evaluated item fit by calculating the S-χ^2^ index to compute the RMSEA values for all items.

## Results

### Confirmatory factor analysis, measurement invariance, and internal consistency

Data analysis was performed on the complete data set which did not have any missing values. No ceiling or floor effects were detected. The CFA confirmed the original unidimensional model of social pain (RMSEA = 0.078; CFI = 0.915; TLI = 0.99(. All 10 item loadings exceeded the minimum threshold of 0.3 suggesting an association with the obtained single factor (see Table 1). This unidimensional model of SPQ demonstrated excellent internal consistency (Cronbach’s α = 0.91). The corrected item-total correlation (CIC) as well as the “alpha if item deleted” index were measured to check the consistency of the remaining items. CICs ranged from 0.58 to 0.73 and the “alpha if item deleted” index indicated no alteration across items.

**Table 1:** Item analysis of the items of the SPQ.

		Item loadings	CICs	Cronbach’s alpha if item deleted
1	It hurts my feelings if somebody denies a request of me.	0.66	0.634	0.905
2	I feel very humiliated when I am excluded from a group.	0.71	0.681	0.902
3	I feel insulted when being ignored at a party.	0.60	0.579	0.908
4	It hurts me when somebody ignores me.	0.75	0.721	0.900
5	When I feel rejected, I experience inner tension.	0.70	0.674	0.903
6	When an acquaintance does not respond to me when I say hello, I feel rejected.	0.75	0.710	0.900
7	When a friend distances himself/herself from me, I feel repulsed.	0.78	0.733	0.899
8	When I get the impression that a colleague withdraws from me, I feel rejected.	0.78	0.727	0.899
9	When somebody declines my request or suggestion, I feel snubbed.	0.72	0.690	0.902
10	If somebody cancels an appointment without a good reason, I feel repulsed.	0.65	0.618	0.906

CIC, corrected item-total correlation.

### Convergent and divergent validity

The zero-order correlations demonstrated convergent validity as well as divergent validity of the SPQ consistent with our expectations. In particular, the SPQ was found to be positively correlated with the IPSM (*r* = 0.636, *p* < 0.001) indicating convergent validity. The IPSM subscales yielded high correlations in interpersonal awareness (*r* = −0.667, *p* < 0.001) and separation anxiety (*r* = −0.553, *p* < 0.001) with moderate correlations with the need for approval (*r* = −0.420, *p* < 0.001), fragile inner-self (*r* = −0.451, *p* < 0.001) and timidity (*r* = −0.391, *p* < 0.001). A moderate negative correlation with the self-esteem scale (*r* = −0.424, *p* < 0.001) and a low correlation with the MSPSS (*r* = −0.161, *p* = 001) were found demonstrating divergent validity. Specifically, low negative correlations with the significant other (*r* = −0.104, *p* = 0.038), family (*r* = −0.197, *p* < 0.001), and friend (*r* = −0.099, *p* = 0.049) subscales of MSPSS were observed.

### IRT

For the item response theory (IRT) analysis, we used the graded response model (Samejima, 1997). CFA supported the assumption of unidimensionality but we tested this assumption further through an EFA using the entire sample. According to the KMO test (KMO = 0.90), this dataset was suitable for EFA. The only eigenvalue was 5.58 which accounted for 55.80% of the variance which supported the unidimensionality assumption for IRT. The S-X2 estimator was computed to assess the fit of polytomous items with *p*-values <0.001 indicating inadequate fit (Kang and Chen, 2008). RMSEA was also used to examine the fit, with values below 0.08 considered good (Bean and Bowen, 2021). Table 2 displays the item fit indexes. All items demonstrated high discriminative power (α > 1.7). The location parameters revealed a consistent rise in all threshold estimators indicating that a higher level of the latent trait was necessary to endorse higher response categories. For instance, individuals with a latent trait score of −1.44 were likely to endorse the first response category (i.e., applies not at all to me) in the first item with at least a 0.6 probability. In contrast, a score of 2.10 was necessary to endorse the last response category (i.e., applies exactly to me).

**Table 2:** Discrimination parameters, location and item fits.

	Item parameters					Item fits			
Items	α	b1	b2	b3	b4	S-X2	df. S-X2	RMSEA.S-X2	*p.* S-X2
1	1.84	−1.44	−0.26	1.02	2.10	50.786	54	<0.001	0.599
2	2.05	−1.21	−0.28	0.59	1.64	62.163	65	<0.001	0.577
3	1.49	−1.59	−0.47	0.53	1.83	60.998	75	<0.001	0.879
4	2.41	−1.44	−0.55	0.29	1.33	57.511	51	0.018	0.247
5	2.09	−1.72	−0.62	0.16	1.28	72.883	57	0.026	0.076
6	2.41	−0.71	0.12	0.81	1.85	36.023	55	<0.001	0.978
7	2.79	−0.90	−0.09	0.50	1.41	58.174	53	0.016	0.291
8	2.75	−0.72	0.11	0.78	1.82	55.456	52	0.013	0.346
9	2.18	−1.01	0.07	0.84	1.94	57.076	56	0.007	0.435
10	1.76	−0.74	0.21	1.02	2.02	58.320	61	<0.001	0.574

**Figure 1:**
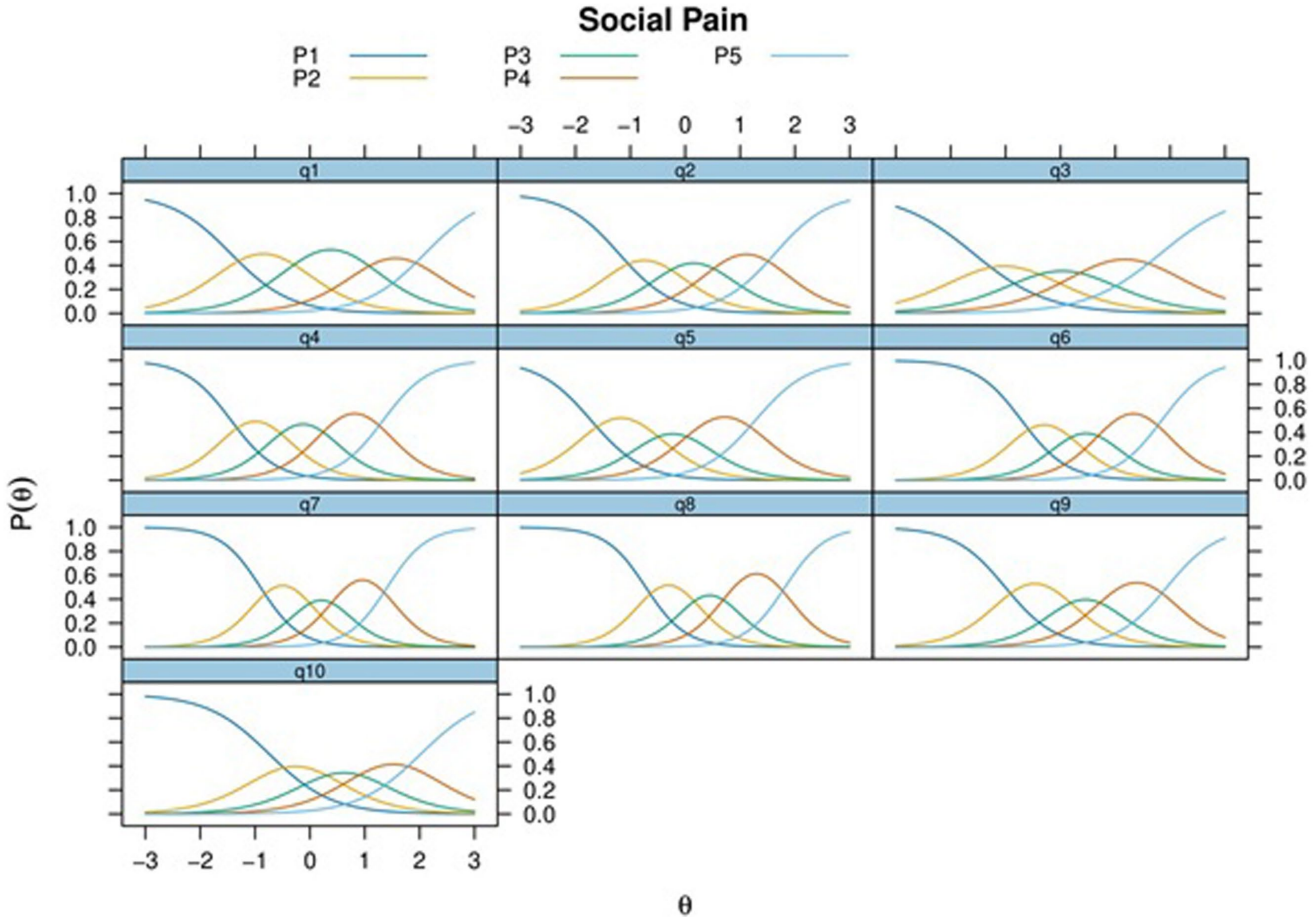
Category characteristic curves for all items of the SPQ.

In Figure 1, the Category Characteristic Curves (CCCs) are depicted for all items. The CCCs reveal that, on the whole, items were endorsed at levels below the average of the latent trait implying lower levels of perceived social pain among participants.

**Figure 2:**
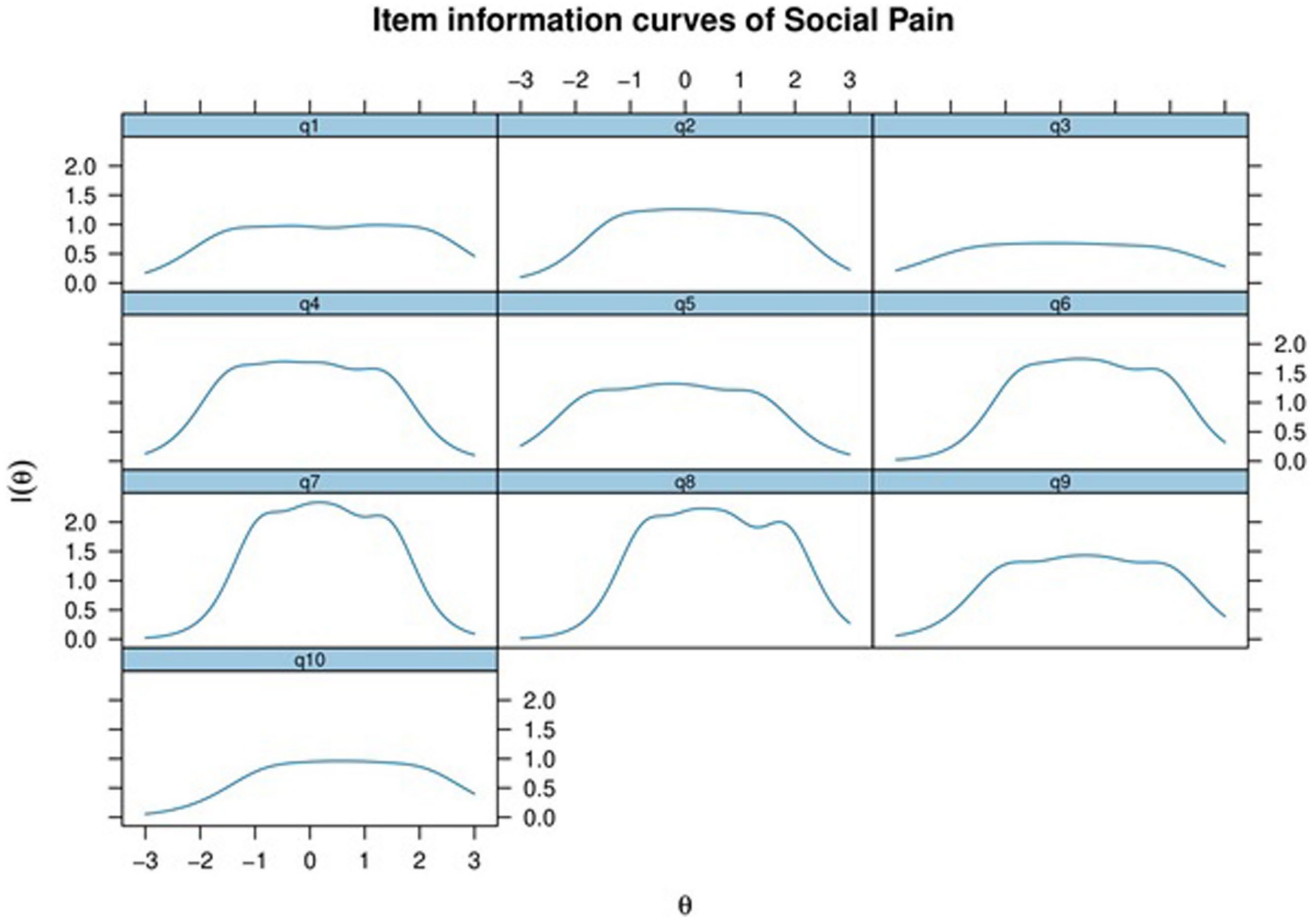
Item information curves for all items of the SPQ.

Figure 2 presents the Item Information Curves (IICs) for all items suggesting that items 7 and 8 provide the most informative contributions. These items contribute more effectively to the precision of score estimation.

**Figure 3:**
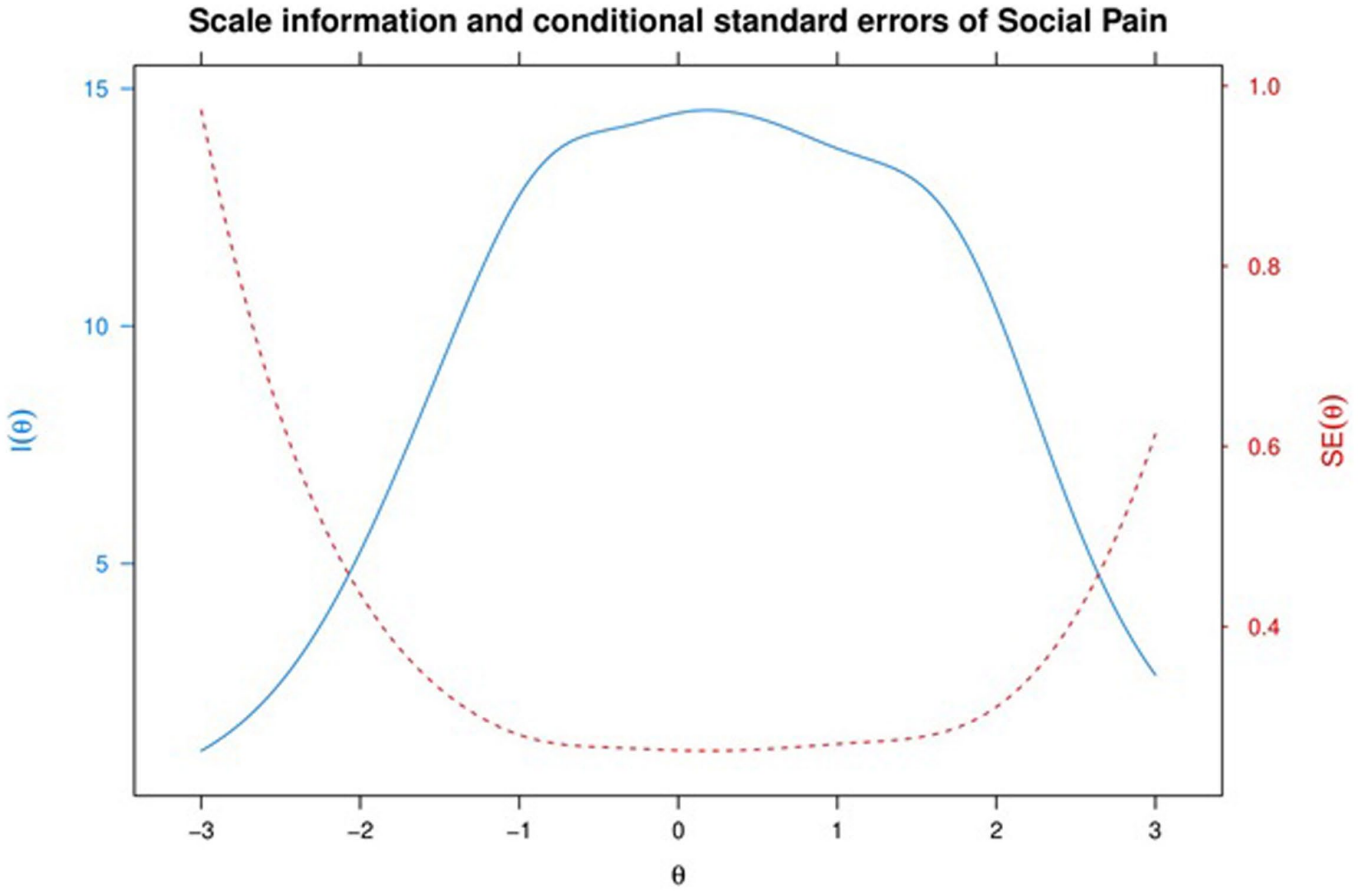
Scale information and conditional standard errors of social pain.

In Figure 3, the Scale Information and Conditional Standard Errors are displayed. The solid line denotes the Scale Information function, while the dashed line represents the Conditional Standard Errors. The location of the peak of the Scale Information function suggests that SPQ provides the most information for theta within the range − 1 to +1.2. By using a Scale Characteristic Curve (SCC), estimated theta scores can be transformed into expected true scores.

**Figure 4:**
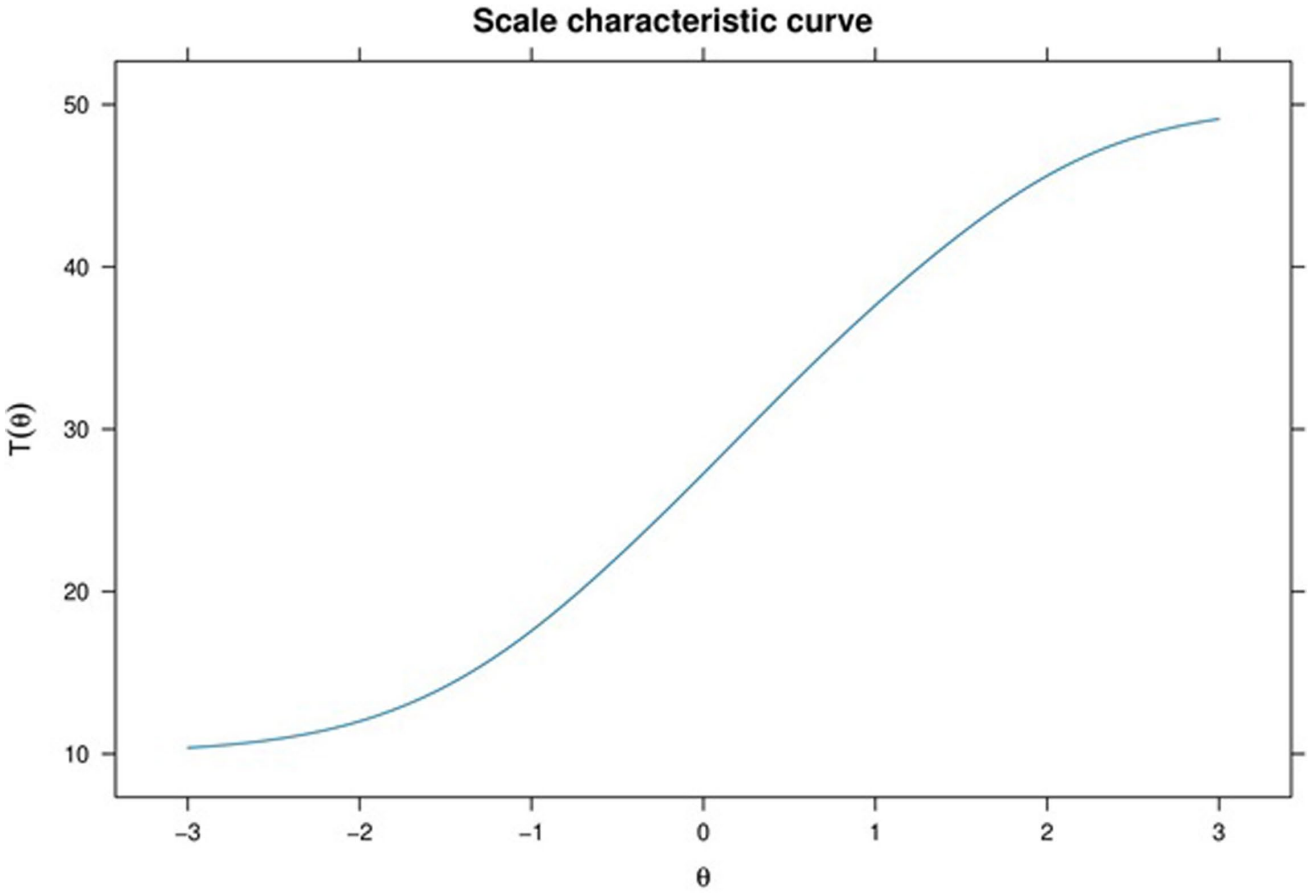
Scale characteristics curve of the SPQ.

Figure 4 displays the Scale Characteristic Curve (SCC) for our sample, enabling us to establish the expected true score corresponding to any given theta score. Note that, reliability in item response theory is defined differently than in classical test theory (i.e., Cronbach’s alpha).

**Figure 5:**
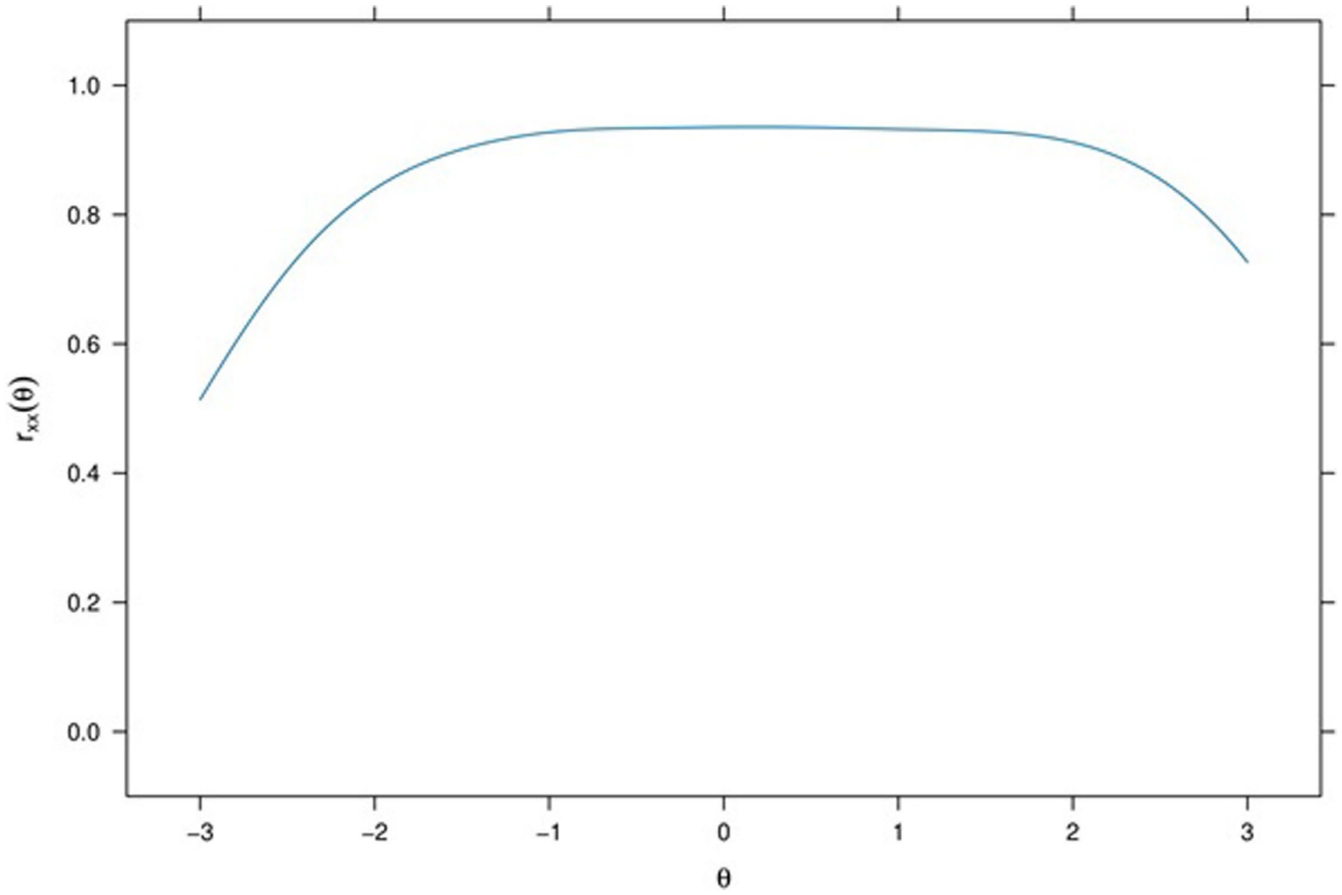
Conditional reliability of the SPQ.

Figure 5 presents the Conditional Reliability. According to this figure estimated scores with theta values ranging from −2 and + 2 demonstrate the highest level of reliability. Finally a marginal reliability level of 0.923 was observed for SPQ indicating high reliability.

## Discussion

Being excluded, rejected, or ignored in interpersonal or intergroup relationships can cause distressful feelings of social pain which are similar to psychical pain and threaten basic needs. The Social Pain Questionnaire (Stangier et al., 2021) serves as the only instrument to assess emotional reactions to social exclusion. In the present study we translated the SPQ into Persian and evaluated its psychometric properties in order to make the SPQ accessible and relevant to the Iranian population. Using the SPQ in a new and different cultural context allows researchers and practitioners to gain a richer understanding of social pain within the Iranian culture, which may have unique characteristics that the original scale does not capture. The Persian version of the SPQ can, with rigorous validation, contribute to both research and practical applications in psychology and psychiatry for assessing social pain in Iran. In addition this is the first study that applies a combination of Item Response Theory (IRT) and CFA to investigate the properties of SPQ which show that its properties are robust. Specifically, our results confirmed the SPQ’s unidimensional factor structure. We also explored correlations of SPQ scores with related external variables in order to provide more information about the validity (convergent and divergent) of the SPQ scores. The results of this study can contribute to enriching the literature on the factor structure, validity, and reliability of social exclusion and ostracism within various contexts. As we discuss below, our findings add to the existing body of the field.

### Factorial structure, measurement invariance, and internal consistency

The findings of our study explored the factorial structure of the SPQ by using exploratory factor analysis. The SPQ involved only one factor, consistent with the original version (Stangier et al., 2021). Each item had high factor loading and communality coefficients for the single factor. Our results of CFA are also consistent with the original version and indicated that the loadings for all other items surpassed the minimum threshold of 0.30 (Brown, 2006) and further supported the unidimensional factor structure of the SPQ with 10 items. Moreover, in line with the initial study by Stangier et al. (2021), the SPQ scores showed high internal consistency (Cronbach’s α = 0.91), demonstrating that the SPQ scores are reliable and that the items measure the same underlying construct.

### Convergent and divergent validity

As expected, results showed high correlation between the Persian version of SPQ and IPSM. In other words, more social pain is related to higher interpersonal sensitivity scores. Preoccupation with social ties and interpersonal relationships for humans is evident in both social pain (Eisenberger, 2012) and interpersonal sensitivity (Boyce and Parker, 1989). As well as social pain, interpersonal sensitivity also includes experiences when one’s self is devalued, rejected or negatively evaluated or when one is sensitive to rejection or criticism (Boyce and Parker, 1989; Eisenberger, 2012).

Our results showed a negative correlation between SPQ and the self-esteem Scale showing that individuals who suffer from social pain have less self-esteem. Self-esteem has a close relationship with interpersonal experiences. According to the sociometer theory (Leary and Terry, 2013) trait self-esteem can be viewed as an indicator of an individual’s general sense of social value and acceptance. It follows that self-esteem levels fluctuate with alternations in social values and real or potential social exclusion. Previous studies showed that self-esteem declines after rejection or exclusion in interpersonal relationships (Leary et al., 1995; Lemay and Ashmore, 2006). Specifically, individuals with high self-esteem have a rich interpersonal experience and high-quality social interactions (Cameron and Granger, 2019). For individuals with lower self-esteem, more activation in dACC and more social pain were found after exclusion. This connection between dACC and the prefrontal cortex (PFC) can explain the negative relationship between self-esteem and social pain (Onoda et al., 2010).

Another negative correlation was found between SPQ and MSPSS indicating divergent validity. This means that higher social pain is associated with lower perceived social support. This finding is in line with previous studies indicating that perceiving inadequate or insufficient social support is strongly tied to social pain (MacDonald and Leary, 2005) and also that support of significant others has a significant role in reducing the negative effects of rejection (Eisenberger et al., 2007; Masten et al., 2012; DiLorenzo et al., 2018). Social support function acts as a self-regulating mechanism to cope with social pain by reducing the automatic dorsal anterior cingulate cortex (dACC) response to exclusion (Eisenberger et al., 2007; Riva and Eck, 2016). Another study also showed that social pain relates to the ventral ACC, and emotional support from others increases prefrontal cortex activity, which can in turn weaken affective responses (Onoda et al., 2009). Furthermore, decreased anterior insula activity [a brain region involved in the processing of negative effects during social exclusion (Singer and Lamm, 2009)] was observed for individuals who experienced exclusion but received emotional social support (Morese et al., 2019). In summary our findings provide further support for the importance of perceiving social support for decreasing automatic social pain signals in the brain as well as increasing regulatory responses (Riva and Eck, 2016).

### IRT

Our data were analyzed using IRT models after establishing the unidimensionality of SPQ and the results demonstrated that across all items the difficulty parameters consistently increased monotonically. To put it more precisely, individuals with low levels of social pain are more likely to choose the first or second response options while individuals with high levels of social pain are more likely to select a higher response option. This pattern of response is expected, as it shows that the provided response choices effectively represent the content of each item. All of the items also showed high discrimination values indicating the tool’s proficiency in effectively distinguishing between the responses of individuals with high social pain and those with moderate or low social pain. This includes the overall assessment of the latent variable expressed in total scores. This scale can provide a more accurate evaluation of social pain among individuals with moderate to high levels of this latent characteristic. In particular, individuals with low or very low social pain are more likely to endorse the first response option than other response options. The discrimination parameters of all items were also high suggesting that the items were effective at separating high and low levels of the latent trait. In summary, the SPQ appears to be a reliable and valid tool for assessing social pain in moderately to severely affected individuals.

### Limitations and future directions

This study contributes to the field of research on social rejection, ostracism and social pain by conducting the first study examining the psychometric properties of SPQ in a non-western society. Additionally, it utilizes IRT models to investigate the unidimensionality of the scale overcoming the shortcomings of classical test theories, however, our findings come with several limitations. First, in the present study, we employed a standard translation process to develop a Persian version of SPQ. Nonetheless, cultural differences between the West (where SPQ was originally developed) and East societies might have a negative impact on our findings. Second, given the various ethnic groups in Iran, it is crucial to consider cultural differences among them as an important factor. Third, the test–retest reliability of the scale has not been examined due to the cross-sectional design of the present study, thus, longitudinal studies are also necessary. Fourth, in recent studies, immigrants have shown links between physical and social pain that are reflective of their adjustment to new cultures (Lu et al., 2017), thus, future research could use SPQ in order to better examine social pain among immigrants. Fifth, future investigations could also explore the relationship between social pain and rejection sensitivity, a relevant concept related to anxiety, a quick perception and overreaction to social rejection (Downey and Feldman, 1996). Finally, in this study, the SPQ was not assessed within the framework of the Cyberball game, which is widely utilized in the social pain research. It may, therefore, be worthwhile to incorporate the SPQ alongside the need-threat scale when examining social pain following rejection in the game.

## Conclusion

To examine the importance of social pain as an emotional experience as painful as the physical experience of pain (Eisenberger, 2015), we looked at the psychometric properties of the SPQ scale in order to contribute to the existing literature, building upon the initial work by Stangier et al. (2021). SPQ was found to be a valid and reliable tool for assessing social pain providing researchers with a valuable instrument to identify individuals experiencing social pain. By employing Item Response Theory (IRT) analysis, we have augmented the theoretical psychometric evaluation of the SPQ scale and demonstrated that SPQ is a unidimensional, valid and reliable measurement tool. Using this approach has led to novel insights that have advanced our understanding of this field. In addition, our study is the first investigation into the utilization of social pain among Iranians. By unveiling usage patterns and behaviors within this cultural framework, we enhance our understanding of how exclusion impacts social pain, thereby contributing to a more comprehensive understanding of this experience.

## Data availability statement

The data supporting the conclusions of this article are available at https://osf.io/g34b5/?view_only=3b44a990c14448c39ba2f090574ef124.

## Ethics statement

Ethical review and approval was not required for the study on human participants in accordance with the local legislation and institutional requirements. Written informed consent from the patients/ participants or patients/participants' legal guardian/next of kin was not required to participate in this study in accordance with the national legislation and the institutional requirements.

## Author contributions

MS: Writing – original draft, Data curation, Formal analysis, Investigation. HF: Writing – review & editing, Methodology, Project administration, Supervision, Writing – original draft. PW: Methodology, Project administration. NA: Formal analysis, Software, Writing – original draft.
